# Global Decline in the Size of Sea Turtles

**DOI:** 10.1111/gcb.70225

**Published:** 2025-05-14

**Authors:** Graeme C. Hays, Mohd Uzair Rusli, David Booth, Jacques‐Olivier Laloë

**Affiliations:** ^1^ Deakin Marine Research and Innovation Centre, School of Life and Environmental Sciences Deakin University Geelong Victoria Australia; ^2^ Institute of Oceanography and Environment Universiti Malaysia Terengganu Kuala Nerus Terengganu Malaysia; ^3^ School of the Environment The University of Queensland St. Lucia Queensland Australia

**Keywords:** Bergmann's rule, body size reduction, *Chelonia mydas*, ectotherm, food availability, macroevolution, responses to climate change

## Abstract

Changes in mean adult body size may be a universal response to global warming and sometimes lead to conservation concerns. We show that size reductions in sea turtles are now the norm and have another explanation. From 18,707 measurements of nester size (curve carapace length) for sea turtles spanning 30 years from Redang Island (Malaysia), where nearly all nesting individuals have been tagged, we show that the mean size was initially fairly stable and then decreased by 4.0 cm from 100.8 cm in 2005 to 96.8 cm in 2022, which likely translates to a change in mean mass from 120 to 105 kg. At the same time, nesting increased from around 300 to 2000 nests per year. Consistent with this finding of a size reduction in an expanding population, at 27 of 31 sites across the globe where changes in the mean size of nesting sea turtles have been assessed, mean size is decreasing, and the most marked decreases are at sites where population size is increasing most dramatically. Taken together, these focal and global findings suggest that an important driver of size reductions in sea turtles is an influx of small first‐time nesters (neophytes) in expanding populations, and hence, size reductions are partially a consequence of successful sea turtle conservation measures and population recoveries. At the same time, the focal observations in Malaysia show that the mean size of neophytes has also been getting smaller over time: from 99.6 to 96.8 cm between 2005 and 2022, likely because of a change in foraging environments. While smaller turtles have lower reproductive output, this negative consequence of decreases in nester size will often be more than offset by increases in nesting numbers that are occurring widely.

## Graphical abstract

Changes in mean adult body size may be a universal response to global warming and sometimes lead to conservation concerns. Using a 30‐year data set for green turtles from Malaysia, we show a decrease in nester length of 4 cm while nester abundance was increasing and similarly found a reduction in nester size at 27 of 31 sites across the globe where changes in the mean size of nesting sea turtles have been assessed. Our findings suggest that an important driver of size reductions in sea turtles is an influx of small first‐time nesters (neophytes) in expanding populations.

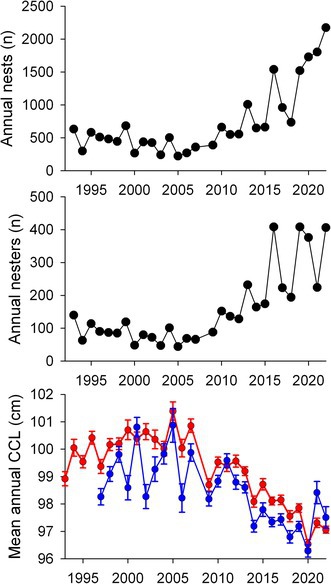

## 1 Introduction

Many taxa around the world are getting smaller in adult body size (Ohlberger 2013). In some cases, decreases in size have been linked to harvesting of larger individuals (e.g., commercial fishing or trophy hunting) (Coltman et al. 2003; Olsen and Moland 2011; Olden et al. 2007). Furthermore, in addition to changes in the range of species and the seasonal timing of events (phenological changes), changes in mean body size may be a universal response to global warming (Gardner et al. 2011). Reductions in size are often attributed to warming temperatures favoring smaller animals and in some (Ohlberger 2013), but not all (Solokas et al. 2023) cases, there is strong empirical support for this hypothesis. For example, reductions in size linked to warming temperatures have been shown in some crustaceans, fishes, amphibians, birds, and mammals (Ohlberger 2013; Genner et al. 2009). In birds, a general response to long‐term warming has been a reduction in body size and increases in bill size to help dissipate more heat, so the shape of birds has changed (Ryding et al. 2021). However, at the same time, occasional very hot years may also lead to reductions in bill size in birds, potentially because those environmental conditions may limit food availability for birds and hence their general growth (Ryding et al. 2024). So even in well‐studied groups, including birds, a full understanding of the reasons underlying changes in mean size remains enigmatic (Solokas et al. 2023). This continued uncertainty of the drivers of changes in body size over time has led to repeated pleas for more regular monitoring of body size, especially for taxa other than marine fish, particularly in conjunction with abundance estimates (Martins et al. 2023).

Sea turtles are a group where, in recent years, decreases in the mean size of nesting turtles have started to be reported at some sites (e.g., Mortimer et al. 2022; Pereira et al. 2023; Evans et al. 2024) although the broader occurrence of this phenomenon has not been assessed. There are two hypotheses to explain this decrease. First, it is known that first‐time nesters (neophytes) are smaller than returning nesters that have nested in a previous year (remigrants) (Mortimer et al. 2022). Where the abundance of nesting turtles is increasing due to an influx of neophytes, then this influx means the ratio of neophytes to remigrants will increase, and so the mean size of nesters will decrease (Mortimer et al. 2022). Second, it has been suggested that the mean size of neophytes might itself be showing a long‐term decline, for example, driven by rising water temperatures and/or a change in foraging conditions (Le Gouvello et al. 2020; Phillips et al. 2021). The two hypotheses are not mutually exclusive and an increase in population size combined with a decrease in the mean size of neophytes will sum together to cause a larger decrease in mean nester size.

Disentangling these two hypotheses is not straightforward, often because at many sites there is incomplete tagging of all nesters. Instead, often only a small proportion of nesting turtles are tagged, and so it is impossible to reliably distinguish neophytes from remigrants. We overcame this challenge by first using a multi‐decadal dataset from Redang Island (Malaysia), where, unusually in sea turtle monitoring, nearly all nesting turtles were observed and identified with numbered flipper tags, and hence, the distinction between neophytes and remigrants can be made reliably. We then conducted a global analysis of the extent of size reductions reported for turtles across the globe and explored the links between size reductions and abundance changes. By this combination of focal and global analyses, we both document the prevalence of size changes in sea turtles across the globe and explain the likely drivers of the changes.

## 2 Materials and Methods

### 2.1 Assessing Changes in Mean Nester Size in Malaysia

Throughout 1993–2022, the body size of nesting green turtles ( *Chelonia mydas* ) was assessed at the Chagar Hutang Turtle Sanctuary (5°48.778′ N, 103°0.502′ E) on Redang Island, Malaysia. The nesting beach was patrolled on foot throughout the night to locate nesting turtles. The monitored area (Chagar Hutang Turtle Sanctuary) remained consistent throughout the entire study period. The beach is approximately 350 m in length, and monitoring was conducted across the full stretch without any change in spatial coverage since the program's inception. The short length of the beach, combined with the fact that green turtles typically take several hours to complete the nesting process and return to the sea, meant that the vast majority (> 95%) of nesting individuals were encountered each year.

Nesting activity was monitored nightly from April to October under the coordination of the Sea Turtle Research Unit (SEATRU), with year‐round presence of at least two trained turtle rangers stationed permanently at the sanctuary. During the nesting season (April to October), the monitoring effort was further strengthened by the arrival of additional personnel, including researchers and trained volunteers, to ensure thorough nightly coverage of the entire beach. Each night, two to four personnel conducted patrols from 20:00 to 06:00 local time, ensuring continuous coverage. The monitoring protocol remained standardized throughout the years, with any changes in personnel numbers not affecting data consistency due to strict adherence to the protocol. Only confirmed nesting events were included in the final dataset. A handful of outlier carapace length measurements, likely transcription errors when data were entered into the database, were removed from the database if they differed by more than three standard deviations from the mean size. Every time a turtle was encountered nesting, the curved carapace length (CCL) was measured and included in the database. Tape measures were calibrated regularly to ensure accuracy in data collection. Calibration was conducted at the start of each nesting season and checked periodically throughout the season to minimize any potential measurement error. All personnel involved in taking morphometric measurements, including CCL, underwent hands‐on training using standardized protocols. This training was conducted directly by the chief scientist overseeing the program during each period: Dr. Chan Eng Heng (1993–2007), Dr. Juanita Joseph (2007–2017), and Dr. Mohd Uzair Rusli (2017–present). So those measuring turtles were either experienced fieldworkers or were trained by experienced fieldworkers. This approach ensured continuity in measurement techniques and consistency in data quality across the years. Each turtle was tagged with Inconel flipper tags on both front flippers or, if already tagged, the tag numbers were noted. If a tag on one flipper was missing, a new tag was attached. There was no change to methodology throughout the time series. The low rate of tag loss at this site means that first‐time nesters (neophytes) can be reliably distinguished from turtles that have nested in a previous year (remigrants) (Nishizawa et al. 2018). Data for the year 2008 were not available owing to failure of the database system.

### 2.2 Literature Compilation of Changes in Mean Nester Size

Using forward and back searches of published papers on changes in the mean size of nesting turtles (e.g., Mortimer et al. 2022; Evans et al. 2024), we assembled the literature from around the world and across species for where changes in mean size have been documented. In the majority of cases, the published change in size was statistically significant. Turtle sizes were usually reported in cm to one decimal point (e.g., 95.8 cm), so to the nearest mm. One paper (Hatase et al. 2002) reports turtle sizes in mm (e.g., 825 mm), which results in the same precision. In some papers, changes in turtle size were reported in tables or in the main text, and in other papers, data were presented in figures. For the latter, we digitized the data using WebPlotDigitizer version 4.7. For each site, we assessed the rate of change of mean size by comparing the mean body size in the last 3 years of each time series (CCL_L_) to the mean body size in the first 3 years of the same time series (CCL_F_) and accounting for the length of the time series (*n* years) using Equation (1):

(1) $$\text{Annual change in body size}=\left[{\left({\mathrm{CCL}}_{L}/{\mathrm{CCL}}_{F}\right)}^{1/\left(n-3\right)}-1\right]\times 100$$

Three years was used in this equation to help remove the impact of inter‐annual variability following procedures used in population trend analyses (Mazaris et al. 2017; Hays et al. 2024). Where available, the change in abundance at each nesting site (change in the number of nests or the number of nesters over time) was obtained from the global syntheses published by Mazaris et al. (2017) and Hays et al. (2024). We extracted population size data for the years that matched the years for which we had found turtle size data. In other words, if we found turtle size data for 1970–2020 at a site, we searched for population size data for the same period of time for that site. The majority (> 90% of studies) of abundance estimates in these previous studies reported the number of nests rather than the number of nesters. Mazaris et al. (2017) converted the number of nesters to the number of nests assuming a clutch frequency of three. The key parameter we extracted from these previous studies was the rate of change of abundance, regardless of whether nests or nesters were considered. The change in abundance was calculated from the mean abundance in the last 3 years of each time series (*N*_L_) compared to the mean abundance in the first 3 years of the same time series (*N*_F_) and the length of the time series (*n* years) using Equation (2):

(2) $$\text{Annual change in abundance}=\left[{\left(N_{L}/N_{F}\right)}^{1/\left(n-3\right)}-1\right]\times 100$$

## 3 Results

### 3.1 Empirical Observation of Change in Size Over 30 Years of Monitoring

**FIGURE 1:**
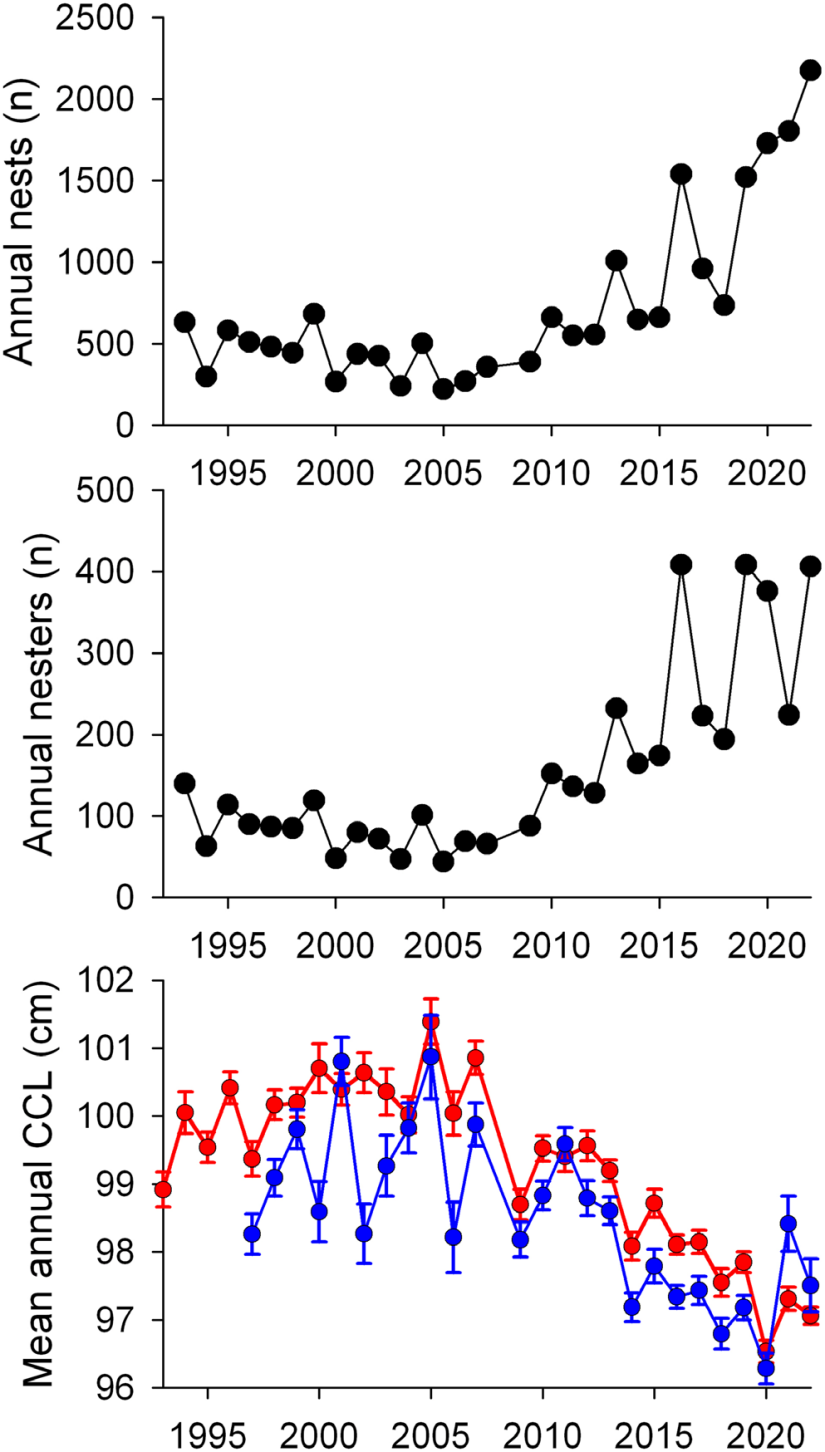
For nesting green turtles at Redang (Malaysia), the change in the annual number of nests (a) and of nesters (b) over 30 years between 1993 and 2022, (c) the change in mean size of all nesters (red line) and of neophytes (blue line). Annual mean CCL is shown as ± 1 SE. From 2005, there was a significant increase in nesting abundance of 13.6% per year, as well as reductions in the mean size of both all nesters and neophytes.

Between 1993 and 2009, the annual number of nests on Redang Island remained relatively constant, before starting to increase markedly in the second half of the time series (Figure 1a). For example, in 2005–2007 the annual number of nests was 221, 267, and 357, which increased to 1727, 1803, and 2174 between 2020 and 2022. This equates to an annual change in abundance of 0.136, that is, a 13.6% increase in abundance per year between 2005–2007 and 2020–2022. The annual number of nesters followed broadly the same pattern as the annual number of nests (Figure 1b).

Over 30 years (1993–2022) there were 18,707 measurements of individual nester size. To examine the difference in size between neophytes/remigrants at the start of the time series, we used data from 1997 up to 2004. Here we selected 1997 as the start year (i.e., year 4 of the project) so that there was sufficient time for individuals to have been tagged, since green turtles typically have a breeding interval of 3 or 4 years at this site. So after 4 years, all the remigrants would have been expected to be encountered and thus untagged nesting turtles were likely neophytes rather than untagged remigrants. Between 1997 and 2004 the mean size of neophytes (mean CCL 99.3 cm, *n* = 1743 measurements, SD = 5.3 cm) was 1.8 cm smaller than that of remigrants (mean CCL 101.1 cm, *n* = 1599, SD = 5.3 cm), a difference that was highly significant (*t*_3306_ = 9.8, *p* < 0.001).

The mean size of nesting turtles followed the reverse pattern to the change in abundance. The mean size of all nesting turtles was initially relatively constant, but then both the mean size of all nesters as well as the mean size of neophytes declined markedly during the second half of the time‐series (Figure 1). For example, for all nesters, for the 15 years from 1993 to 2004 the mean CCL was 100.0 cm (*n* = 5108 measurements, SD = 5.3 cm). Then from 2005 to 2022 the fitted line of the linear decline in mean annual CCL (*F*_1,15_ = 96, *p* < 0.001, *r* ^2^ = 0.86) indicated an annual decline in CCL of 0.23 cm and a decline from 100.8 cm in 2005 to 96.8 cm in 2022, that is, 4.0 cm or 4% over 17 years. Over this same period (2005–2022) of the linear decline in the mean CCL of all nesters, the mean annual CCL of neophytes also decreased significantly (*F*_1,15_ = 18, *p* < 0.001, *r* ^2^ = 0.55) with the fitted line showing a decrease from 99.6 to 96.8 cm, that is, 2.8 cm, over this timeframe.

If there was just an influx of neophytes, then the mean annual size would only have decreased by a maximum of 1.8 cm (the difference in mean size of neophytes vs. remigrants at the start of the time series), that is, 45% of what was observed. If only a decrease in size of neophytes was occurring, then the mean annual size would decrease by a maximum of 2.8 cm, that is, 72% of the observed decline. So, the observed decline in mean size is likely driven by a combination of both the influx of neophytes and that the neophytes are getting smaller each year.

### 3.2 Global Patterns in the Change in Mean Nester Size

**FIGURE 2:**
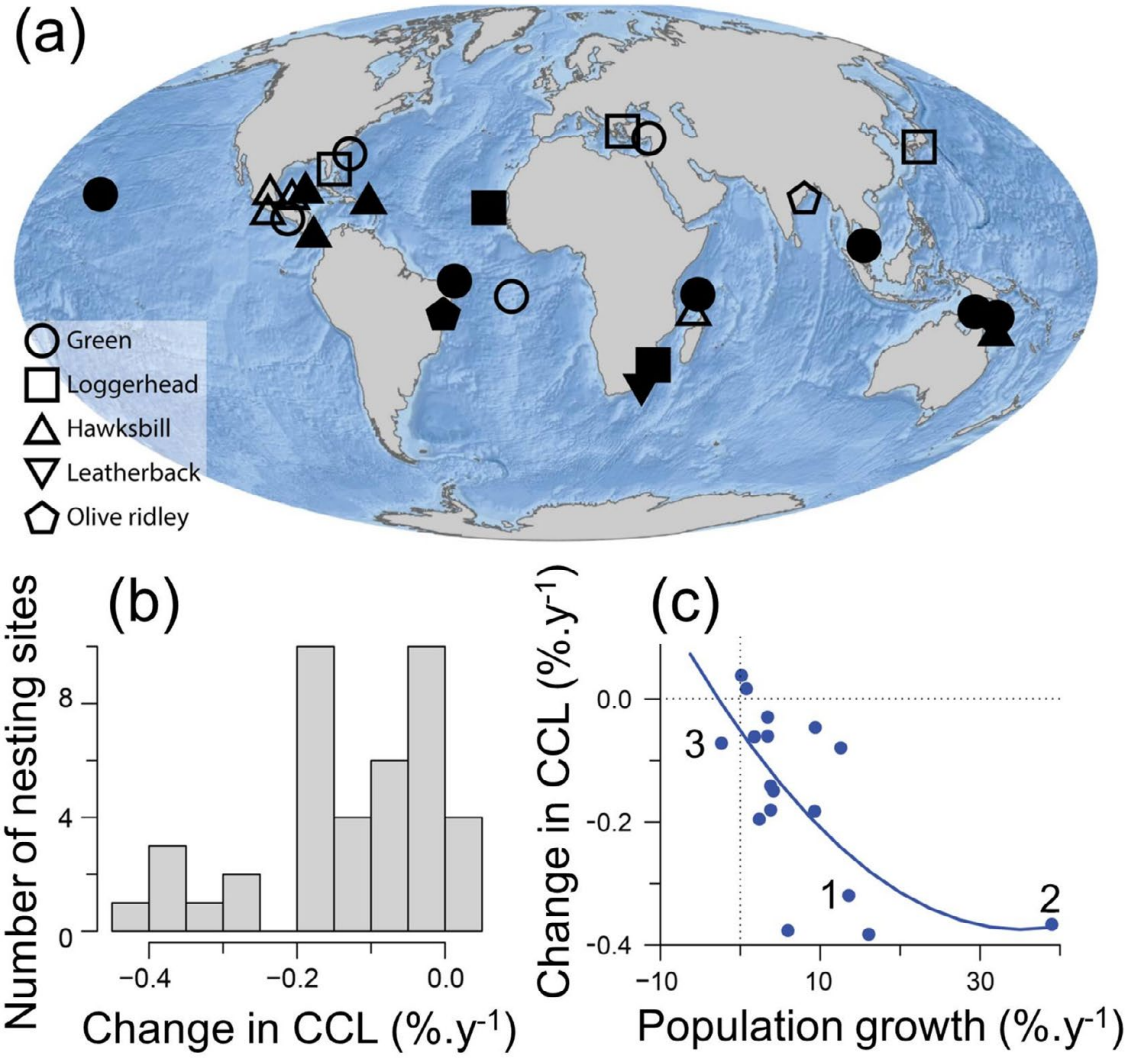
(a) Sites around the world where trends in the mean size of nesting turtles have been reported. Filled symbols indicate sites where both changes in mean size and nesting abundance were available. Open symbols indicate sites where only changes in mean size were available. Different symbols (e.g., circles and squares) indicate different species. (b) The change in mean nester size reported at sites around the world. Most data reports simply mean size of nesters, but in a few cases, the change in the mean size of remigrants and neophytes is both reported, and in these cases, there will be two points for one site. In one case (i.e., olive ridleys nesting in Orissa, India), mean sizes were reported for males and females separately. We removed males from our analysis. Including males in the analysis did not change the results, since males and females had a very similar decrease in size (decrease from 72.9 to 68.8 cm for males and a decrease from 72.6 to 68.6 cm for females over the same period of time), but since data from other sites are for females only, we did not include the males in our analysis. In only four cases reported around the world was there an increase in mean nester size, and in all these cases, the increase was very small. (c) The relationship between the rate of change of mean nester size (% per year) versus the rate of change in nesting abundance (% per year). All the decreases in mean size found in published papers were statistically significant. In general, the most marked decreases in mean turtle size are observed at sites with the most marked increases in population size. The line represents the fitted quadratic curve (*F*_2,14_ = 6.081, *p* = 0.0126, *r* ^2^ = 0.464). The dotted lines indicate the origin for each axis (i.e., *x* = 0 and *y* = 0). Point 1 indicates Redang Island (current study); point 2 represents Sal, Cape Verde, which has the highest reported rate of population growth; and point 3 represents Milman Island, Australia, where a decrease in population size was reported over 28 years. Data compiled from: This study, Bjorndal et al. (1985), Hatase et al. (2002), Limpus et al. (2003), Shanker et al. (2004), Ilgaz et al. (2007), Da Silva et al. (2007), Pérez‐Castañeda et al. (2007), Bellini et al. (2013), Tucek (2014), Weber et al. (2014), Balazs et al. (2015), Piacenza et al. (2016), Sönmez (2019), Bell et al. (2020), Le Gouvello et al. (2020), Phillips et al. (2021), Gulick et al. (2022), Hays et al. (2022, 2024), López‐Castro et al. (2022), Mortimer et al. (2022), and Evans et al. (2024).

Across the globe, there were 31 nesting populations where a change in mean nester size has been reported, with these datasets generally occurring since 1990 (Figure 2a). These data included 13 nesting sites for green turtles, 9 nesting sites for hawksbills, 6 nesting sites for loggerhead turtles, 2 nesting sites for olive ridleys, and 1 nesting site for leatherbacks. At 27 of these 31 sites, the mean nester size has decreased (Figure 2b). This decrease averaged 0.14% per year (range 0.01%–0.38%, SD = 0.12%, *n* = 31 sites). Where changes in mean size were also partitioned between remigrants and neophytes, both groups showed decreases in size. Where there were data on changes in nesting abundance across the same years as data on changes in mean nester size, there was a significant relationship between the rates of change of body size and nester abundance (Figure 2c). The maximum rate of increase in population size (around 39.0% per year) was reported for loggerhead turtles nesting in Cape Verde, which also had the third maximum rate of decrease in mean body size (mean size has decreased from around 83.4 cm in 2009–2011 to 80.7 cm in 2018–2020). A decrease in population size was only observed at one site, that is, Milman Island, Australia, which hosts a hawksbill population. Despite the reduction in population size, the mean turtle size has still decreased at this site, albeit at a slow rate (0.07% per year) (Figure 2c).

## 4 Discussion

Sea turtles face a number of threats, for example, due to climate change, fisheries bycatch, and pollution (Tomillo et al. 2012; Tomillo 2024; Fuentes et al. 2023; Mazaris et al. 2023; Seminoff 2024). Reductions in mean size at maturity are often linked to harvesting in other taxa. For example, overfishing may remove large individuals and lead to reductions in size at maturity (de Roos et al. 2006). In one well‐known example, cod ( *Gadus morhua* ) in the Northwest Atlantic, the collapse of the stock between the mid‐1980s and mid‐1990s was linked to the size at maturity decreasing by about 1 cm per year (Olsen et al. 2004). With trophy hunting (e.g., mammals with big horns or antlers), harvesting may remove large individuals, leading to a decrease in mean size (Coltman et al. 2003). However, the decreases in the mean size of sea turtles are not linked to harvesting but instead quite the reverse. Significant harvesting of some populations of two sea turtle species, green turtles for meat, and hawksbill turtles for shell occurs, but in both cases, there is no evidence that larger females are being targeted (Rahman et al. 2024). Indeed, apparent indigenous hunting of green turtles in the northern Great Barrier Reef region has led to a decrease in the abundance of adult turtles of all sizes (Bell et al. 2025). Across the globe, conservation measures operating at local, national, and international levels have led to widespread increases in nesting numbers across species (Mazaris et al. 2017). These increases in abundance are observed in all sea turtle species, but with leatherback turtles, widespread declines in abundance continue to occur at some sites (Hays et al. 2024). Our evidence from both an intensely sampled focal nesting site as well as a global analysis shows that increases in nesting numbers lead to the most marked reductions in mean nester size, likely due to an influx of first‐time nesters, which are smaller than established nesters. In this sense, a marked reduction in mean size can be viewed as a positive conservation outcome as it reflects an increase in first‐time nesters, even though smaller first‐time nesters have slightly lower reproductive output than established nesters (Wu et al. 2022).

The changes in mean carapace length that we report might, at first glance, seem small, but likely translate into substantial changes in body mass over extended periods. For example, in Malaysia, we report a 4% reduction in mean carapace length for nesting green turtles over 17 years: from 100.8 cm in 2005 down to 96.8 cm in 2022. Using the relationship between carapace length and body mass in nesting green turtles (mass [kg] = 3.75 CCL—258, Hays et al. 2002), this reduction in mean carapace length likely equates to a reduction in mass from 120 to 105 kg, that is, a reduction of 12.5%. Such size reductions will have wider life history implications. It has been argued that the lower reproductive output of smaller turtles might counterbalance increases in nest numbers (Le Gouvello et al. 2020). However, this is likely often not the case. For example, the number of nests in Cape Verde has increased from 506 to 35,507 nests between 2008 and 2020, while the mean size of nesters has decreased by about 2.4 cm, from 83.2 to 80.8 cm (Hays et al. 2022). Using the known scaling of clutch size with body size (Hays and Speakman 1991), the mean clutch size would be expected to decline from 119 to 112 eggs per female over this period. So, then the change in egg production would be from about 60,000 to 4 million eggs per year between 2008 and 2020, that is, the increase in nest numbers far outweighs any decrease in mean clutch size. Similarly, in Malaysia, a slight reduction in mean clutch size due to the decrease in mean nester size will be far outweighed by the sevenfold increase in nesting numbers.

A second clear finding is that the mean size of neophytes is also decreasing. These data are rarely available because not all nesting turtles are generally tagged, making it nearly impossible to distinguish neophytes from remigrants. This decrease in the size of neophytes is harder to explain equivocally. Possibilities include a direct impact of rising sea temperatures on size at maturity, as has been noted in some other taxa (Sheridan and Bickford 2011), climate change negatively impacting habitat quality, or density‐dependent declines in food availability as turtle numbers increase (Phillips et al. 2021). Teasing apart these alternative explanations is not straightforward. Likewise, in some birds, one proposed explanation for a decrease in mean annual size in populations that are increasing in abundance is overexploitation of food (Cooch et al. 1991).

Tag loss at the study site in Malaysia has been estimated to be relatively low (Nishizawa et al. 2018). For example, the probability of a turtle losing both flipper tags within 4 years of being tagged was estimated at 0.05 for Inconel tags (Nishizawa et al. 2018). Likewise, tag loss occurs at sea turtle study sites around the world. A consequence of tag loss is that a small proportion of turtles returning as remigrants (return nesters) will be misidentified as neophytes (first‐time nesters) because they have lost both tags. This mis‐identification may lead to an underestimate of any differences in the size of neophytes versus remigrants. However, in Malaysia, there were clear differences in the size of neophytes versus remigrants, suggesting this difference was not blurred by tag loss. Across sea turtle studies, it is rarely reported explicitly if tape measures are regularly calibrated. However, the flexible tape measures used to measure curved carapace length are cheap and so are often frequently replaced in field programmes, and there is no reason to expect their calibration to change appreciably (e.g., due to shrinkage or stretching of the tape measure). Nevertheless, in future studies, recalibration of tape measures would be useful to confirm the consistency of measurements across years for sea turtle projects around the world (Bolten 1999). We note also that when assessing trends in turtle abundance, the number of nests and the number of individuals might conceivably not follow the same trend, for example, if there is a real change in clutch frequency across nesting success. However, this was not the case at Redang Island, where the annual number of nests and the number of nesters followed broadly the same pattern. However, clutch frequency is often not assessed accurately by the traditional approach of patrolling beaches on foot (e.g., Esteban et al. 2017). Moving forward, the annual number of nests is likely the easiest and most consistent metric to use when reporting population trends across the world.

Our findings show how some of the hypotheses to explain the decrease in mean size of nesting turtles (Mortimer et al. 2022) can be disentangled. Given the empirical evidence for both (i) increases in the proportion of neophytes and (ii) decreases in the size of neophytes impacting the mean nester size, we can conceptualise the drivers of the magnitude of mean nester size reductions reported across the globe. Where nester abundance is not changing and so the proportion of remigrants is not changing, then all else being equal, we expect smaller reductions in mean nester size, driven only by any reduction in the size of neophytes. Examples of smaller reductions in mean size occurring where population size is changing less rapdily include green sea turtles nesting in Aldabra Atoll, Seychelles (Mortimer et al. 2022) and leatherbacks nesting in iSimangaliso Wetland Park, South Africa (Le Gouvello et al. 2020). However, when both neophytes are getting smaller and populations are increasing much more rapidly due to an influx of neophytes, then we expect the largest reduction in mean nester size, such as those we found for green turtles in Malaysia and the large reduction in size reported for loggerhead turtles in Cape Verde (Hays et al. 2022).

In conclusion, we show that decreases in the mean size of sea turtles are a nearly universal feature at nesting sites around the globe, often being particularly marked where the nest abundance is increasing. As such, a decrease in mean nester size can often be viewed as a positive conservation outcome reflecting population recoveries. However, changes in population size only explain a minority (*r* ^2^ = 0.46) of the variation between sites in the rate of mean size decrease. This finding implies that other factors are also important drivers of the changes in mean size, such as factors driving the change in size of neophytes. There may also be differences between species linked to varying climate change impacts on their different food availability. Sea turtles differ in their diet, with, for example, green turtles tending to feed on seagrass and algae (Fuentes et al. 2006; Esteban et al. 2020) while leatherbacks feed on gelatinous zooplankton (Houghton et al. 2006). Climate change might be expected to impact these different food types differently. The cumulative impact of local threats might also differ between species and populations (e.g., illegal and traditional harvesting), potentially influencing differences in mean size trends around the world. Dynamic energy models that attempt to explain energy balance and how individuals apportion energy to growth and reproduction (e.g., Stubbs et al. 2019) might also shed further light on the reasons for different trajectories in mean turtle size across sites. Longer‐term monitoring of changes in abundance and body size might also reveal further details of the drivers of body size changes and if and how mean body size continues to change when nester numbers stabilize. For example, as neophytes become remigrants and continue to nest, they also continue to grow. So, one prediction is that following an influx of neophytes in an expanding population, the mean size may eventually, after some years, start to rebound and increase as those neophytes continue to grow.

## Author Contributions

**Graeme C. Hays:** conceptualization, formal analysis, investigation, methodology, project administration, writing – original draft. **Mohd Uzair Rusli:** data curation, investigation, methodology, project administration, writing – review and editing. **David Booth:** investigation, methodology, writing – review and editing. **Jacques‐Olivier Laloë:** data curation, formal analysis, investigation, methodology, visualization, writing – original draft.

## Ethics Statement

The survey was approved by the Universiti Malaysia Terengganu Research Ethics Committee (UMT/JKEPHT/2018/23).

## Conflicts of Interest

The authors declare no conflicts of interest.

## Data Availability

Data that support the findings of this study are available from Dryad at https://doi.org/10.5061/dryad.fqz612k41.
